# Diseases of Throat and Lungs

**Published:** 1853-11

**Authors:** 


### *Diseases of the Throat and Lungs.*

I think it was in March, 1838, that Dr. T. K. Chambers, of London, published in the *Lancet*, and also in the *Medical Gazette*, an account of his use of an *inhaling powder*; and giving its *composition*, I immediately had some of it prepared according to his formula, which is as follows:—

"The plan is inhalation of a light innocuous powder, which may carry with it the required substance, either diffused in the air or absorbed in its pores.—That which I found well suited to the purpose is the pollen of the lycopodium, or club mess, which has been made to imbibe as much as it would take up of a saturated solution of nitrate of silver, or of sulphate of copper, or of the two combined, and then carefully dried, and reduced gain to an impalpable powder.

I found this powder servi able in several cases of bronchitis, laryngittis, ulcerated sore thro t, inflammation of the mucous follicles, and in incipient phthisis. It is much preferable prepared as here directed, to that mixed with sugar, as the *real pulverized nitrate* was then used; but as here prepared, the *nitrate* is first *dissolved* in pure water, then the "pollen of the moss" is dipped in a saturated solution, (or that of any other strength desired,) then dried, and *finely pulverized*. It can be of any desirable strength, and should contain less of the *nitrate* that than made from a *saturate* solution, when employed with very irritable patients.

The caustic and astringent property of the nitrate is often useful in chronic catarrh, or in a recent cold, by combining a little of this powder with any kind of snuff, or snuffing the simple powder. It can also be conveniently applied to *indolent* ulcers, and answers every purpose of the stick and lunar caustic or solutions of it. It is useful, also, in various cutaneous diseases, such as ringworm, nettle rash, &c. I had one case of *tetter*, which was cured by it, after resisting numerous other remedies.

A small quantity, say three or four grains of the powder, is put into the receiver of the inhaler the inhaler is then placed in the mouth of the patient, as far back upon the tongue as can be conveniently borne; then held by the lips, or left hand of the patient, while with the right hand the receiver is twirled round to scatter the powder, and by a full inspiration at the same time is is conveyed into the throat. This process may be repeated once a day, or more frequently, if desirable. If the solution is used, the shower syringe is altogether more convenient and easy of application, and agreeable both to practitioner and patient, and does the work much more thoroughly than the probang.

If the mucous membrane of the pharynx or larynx is inflamed, or very tender, and the powder contains the full strength of a saturated solution of the nitrate, it will produce slight smarting or tingling in these parts I have used it in canker of the throat, in what has been called clergyman's sore throat, and in chronic mucous inflammation of any portion of the air-tubes, with much satisfaction—its specific effects being soon perceived. In ulcerated sore throat, I think it the best remedy that can be used. In *bronchitis*, it does what I think no other remedy will do so well, causing a speedy clearing of the tubes, and disposing them to take on a healing action. In phthisis, I cannot speak so confidently of its success, though, in its first stage, I have often, and once or twice in the second stage, seen it prove very serviceable. I have sent it, with an inhaler, to more than thirty physicians in the country, from many of whom favorable acconnts have been returned. Some have found it so successful that they have sent, at many different times, for more of it. Some have said that they have raised patients with it who they felt confident counld not have been raised by former modes of treatment.

I have also made trial of the zinc, copper, and some other astringents, prepared in the same way; but I think the nitrate, for general use, is preferable to any other. The sulphate of copper, in some cases, has been as serviceable, and I have thought, even more so, in syphilitic sore throat.

In a class of diseases which have so very generally resulted in death, it seems to claim the attention of medical men, and to deserve a fair and thorough trial.—*Dr. Cornell's Boston Medical and Surgical Journal.*

